# The effect of metformin therapy on serum thyrotropin and free thyroxine concentrations in patients with type 2 diabetes: a meta-analysis

**DOI:** 10.1038/s41598-023-43266-9

**Published:** 2023-10-31

**Authors:** Alireza Amirabadizadeh, Atieh Amouzegar, Ladan Mehran, Fereidoun Azizi

**Affiliations:** Endocrine Research Center, Research Institute for Endocrine Sciences, Shahid Beheshti University of Medical Sciences, Tehran, I.R. of Iran

**Keywords:** Biochemistry, Diseases

## Abstract

Type 2 diabetes and thyroid function disorders are two common chronic endocrine disorders with the high prevalence in various populations. Metformin is well established as the first-line drug therapy for managing diabetes mellitus. In this meta-analysis, we aimed to determine the effect of metformin on serum TSH and FT4 concentrations in patients with type 2 diabetes. We searched PubMed, Scopus, web of science, Cochrane library, and google scholar to collect information on the effect of metformin on serum TSH and FT4 levels. Demographic and clinical information and serum TSH and FT4 concentrations before and after metformin treatment were extracted. Studies on patients over 18 years of age were included. A total of 11 studies including 1147 patients were selected for the final analysis. In hypothyroid patients, the TSH level decreased significantly after treatment with metformin (Hedges’s g:1.55, 95%CI 0.93–2.16, *p*-value < 0.001); FT4 level increased slightly after taking metformin, but the increase was not significant (Heddges’s g: − 0.30, 95%CI  − 0.90,0.31, *p*-value = 0.34). In euthyroid subjects, the slight decrease found in TSH and FT4 concentrations was not statistically significant. Metformin reduces TSH levels in hypothyroid patients; however, it has no effect on TSH levels in euthyroid patients. Metformin does not affect serum FT4 levels in euthyroid and hypothyroid patients.

## Introduction

Type 2 diabetes (T2D) and thyroid function disorders are two common chronic endocrine disorders with the high prevalence in various populations. Diabetes and thyroid disease are two closely related disorders. The NHANES III study found a higher prevalence of thyroid disease among people with diabetes in the United States than those without diabetes, particularly among patients with positive anti thyroperoxidase antibodies^1^. The incidence rate of clinical hyperthyroidism in women and men was 1.4 and 0.21 per 1000 people per year, respectively^2^. The crude annual incidence rate of type 2 diabetes was 9.94 per 1000 people per year^3^. Thyroid disorders in the general Indian population is estimated to be 10%, while its prevalence in patients with diabetes is 24.8%^4^.

Metformin is well established as first-line drug therapy for managing T2D with a good safety profile. This biguanide acts as an insulin sensitizer in the liver by inhibiting hepatic gluconeogenesis, activating AMP-activated protein kinase (AMPK), and enhancing glucose uptake in the skeletal muscle^5,6^. Multiple studies have reported diverse changes in serum TSH and free thyroxine (FT4) levels following metformin administration in patients with T2D over the past decade. Regarding the effect of metformin on thyroid function, several hypotheses have been proposed. Metformin can activate the TSH receptor by changing the quantity and affinity of thyroid hormone receptors, augmenting the effects of thyroid hormones in the pituitary gland^7^. The highest concentration of metformin is found in the pituitary gland, where it crosses the blood-brain barrier. It inhibits the activity of AMPK in the hypothalamus, despite its activation in the periphery. As a result, the effect of thyroid hormones on the pituitary gland is amplified, resulting in the suppression of TSH^8,9^.

The findings of a few studies have shown a decrease in serum TSH levels in people treated with metformin, however the findings in diabetic patients having hypothyroid or euthyroid function have been a matter of controversy.

No meta- analysis has been conducted to assess the effect of metformin on thyroid status in hypothyroid and euthyroid diabetic patients up to now. As diabetes and hypothyroidism are common endocrine disorders which might occur together, it is important to address the knowledge gap about how to manage thyroid dysfunction in diabetic patients especially in the elderly. Therefore, we conducted a meta-analysis to determine the effect of metformin on serum TSH and FT4 indices in hypothyroid and euthyroid diabetic patients.

Diabetes and hypothyroidism are common hormone disorders that can occur together. It is important to fill the knowledge gaps about how to treat thyroid problems in older people with diabetes.

## Materials and methods

### Search strategy

We searched online databases including PUBMED, SCOPUS, Web of Science, Cochrane library, and Google Scholar from December, 1 2000 until November,1 2022 in using following keywords: metformin, thyroid OR "thyroid dysfunction" OR "thyroid-stimulating hormone” OR “thyroid hormone” OR hypothyroidism OR hyperthyroidism OR thyroxin OR “free thyroxin”, "diabetes mellitus" OR "type 2 diabetes". We searched for the key terms in the title, abstract, and keywords. There were no language restrictions in the extract of the articles. We provide details on the searching method in the supplementary appendix.

### Eligibility criteria

In this meta-analysis, we included patients with T2D who received metformin treatment (with or without levothyroxine) and their TSH and/or FT4 were reported before and after metformin administration. The studies with at least ten adult participants aged > 18 years and under metformin treatment whose TSH and FT4 levels were reported before and after the intervention were evaluated. We excluded the studies on patients with type 1 diabetes.

### Selection of study

Two authors independently extracted the data from the articles, fulfilling inclusion and exclusion criteria, and entered them in the designed form. Any disagreement was resolved with the consensus of the third author. If no information about the articles was available, the researchers emailed the authors to get information. We extracted the following information from each study and tabulated it. Author name, year of publication, sample size, sex and mean age of the patients, dose of metformin, levothyroxine treatment status, the dose of levothyroxine, baseline serum TSH and FT4 concentrations, and treatment duration. We evaluated the clinical trial studies with the Cochrane risk of bias instrument. Newcastle–Ottawa Scale instrument was used for cohort studies.

### Definition

Normal range of TSH 0.35-5.5mIU/L and fT4 11.5-23pg/mL was reported.

### Statistical analysis

Statistical analysis of before/after TSH and FT4 changes (95% CI) was performed with STATA 17.0. We showed each outcome's combined hedges’s g, in a random effect model with a forest plot. We evaluated the heterogeneity between the studies with the I^2^ index. I^2^ values of 0% indicate no heterogeneity, 25% low, 25% to 50% moderate, and 50% high heterogeneity. Publication bias was analyzed by the begg test and presented as a funnel plot. To assess the effect of baseline characteristics of patients, including sample size, mean age, duration of treatment, dose of metformin, and levothyroxine dose, we performed a meta-regression analysis.

## Results

**Figure 1:**
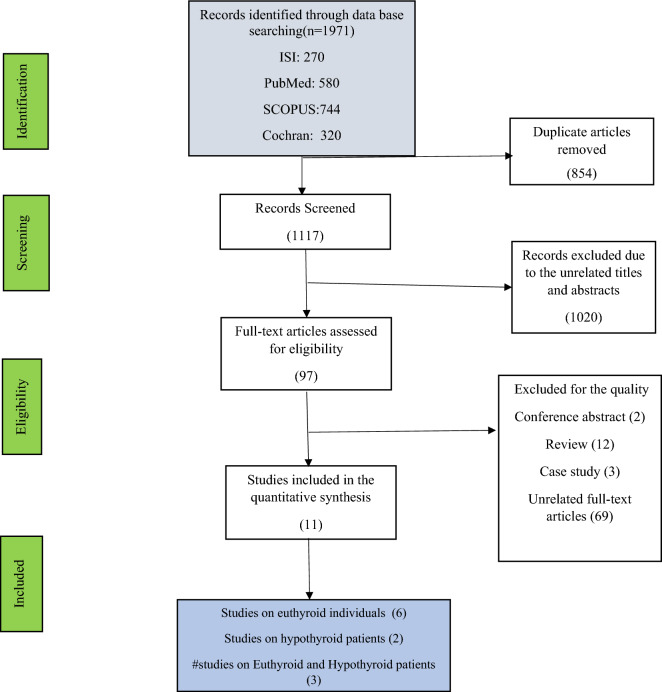
Flow diagram demonstrating the study selection for meta-analysis. # Three studies were performed on hypothyroid and euthyroid patients.

After searching the databases and removing duplicate results, we screened 1117 articles based on the title, and 97 articles were selected to review the abstract and text. Finally, 11 studies were evaluated for meta-analysis, including six studies on euthyroid patients, two on hypothyroid patients and three on both euthyroid and hypothyroid patients (Fig. 1).

**Figure 2:**
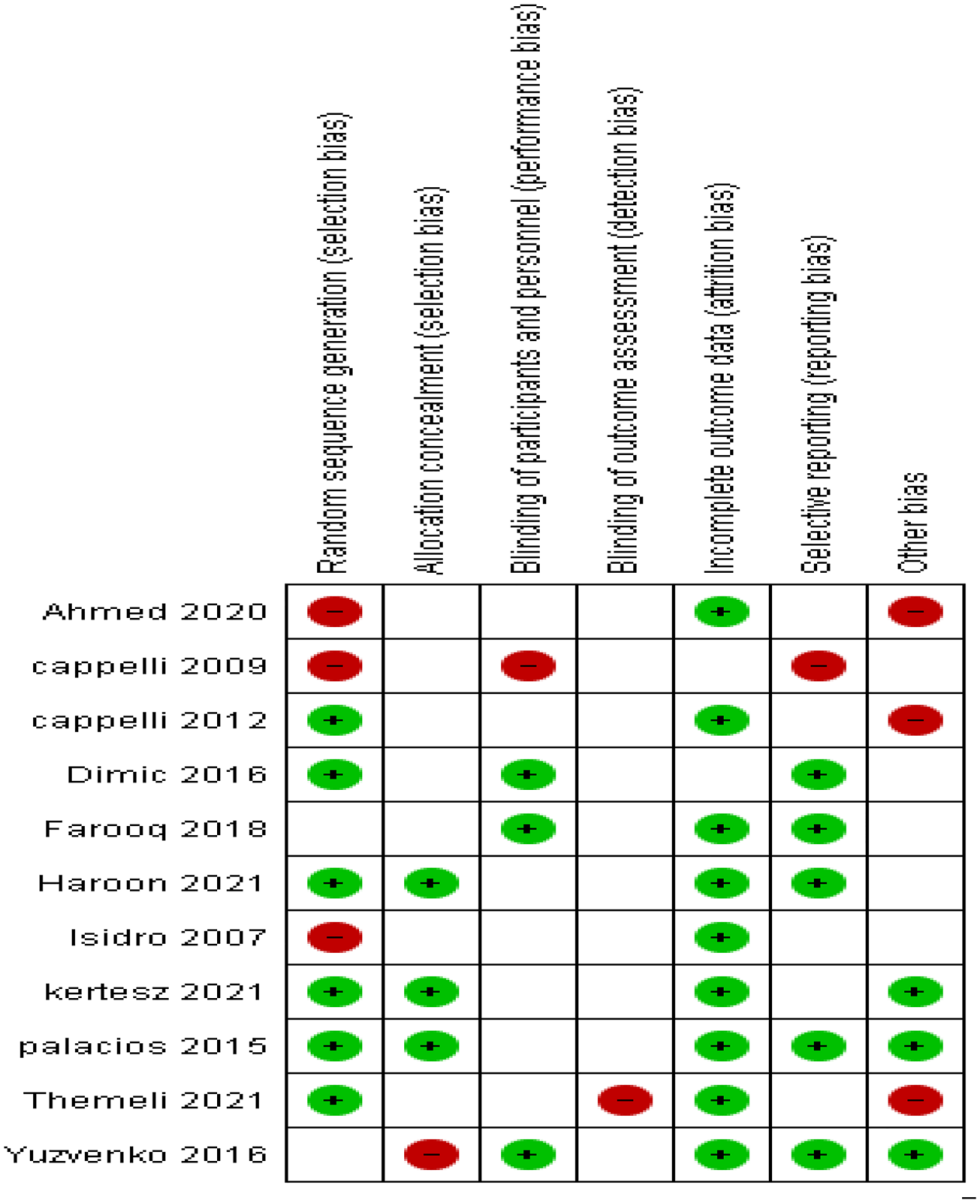
Risk assessment bias for the meta-analysis.

We assessed bias risks for the studies (Fig. 2). Six studies^10–15^ with acceptable methods for randomization and three studies^10,11,14^ with allocation concealment, rated as low risk of selection bias, while five studies reported insufficient information and were categorized as unclear risk. In addition, we rated seven trials^10–12,14–17^ as unclear risk and three studies^13,18,19^ as low risk of bias for blinding participants, personnel and investigators. Ten trials had unclear risk of detection bias. We rated nine trials as unclear risk of attrition bias. In addition, we rated five trials^10,13,14,18,19^ as having a low risk of reporting bias and three other trials^12,15,16^ as having a high risk.

### Euthyroid patients

#### Effect of metformin treatment on TSH

Among nine studies on euthyroid patients; seven^10,11,13,16,18–20^ were clinical trial, and two^14,15^ had retrospective design. Duration of metformin use was less than six months in six studies and between 6 and 12 months in three studies. One thousand one hundred forty-seven people in the metformin group were evaluated. The mean age of the subjects was 54.89 ± 9.69 years. The mean serum TSH concentration was 2.38 ± 0.93 and 2.27 ± 0.90 mIU/L before and after intervention, respectively (Table 1).

**Table 1:** The characteristics of euthyroid patients in the studies at the baseline and follow-up.

Author, year	Haroon, 2021^10^	Kertész, 2021^11^	Ahmed, 2020^16^	Farooq, 2018^18^	Dimick, 2016^13^	Yuzvenko, 2016^19^	Palacios, 2015^14^	Cappelli, 2012^15^	Cappelli, 2009^20^
Sample size	100	21	40	229	170	52	278	203	54
Male/female	Both	Both	Both	Both	NR	Both	Both	Both	NR#
Age (y) mean ± SD	45.2 ± 7.8*	55 ± 12	54 ± 9.5	52.29 ± 7.80	NR	NR	70.23 ± 11.39	54.1 ± 11.2	NR
Bassline TSH (mU/L)	3.95 ± 0.60	2.2 ± 1.2	1.46 ± 0.76	2.69 ± 0.41	2.54 ± 0.97	2.73 ± 0.81	2.00 ± 0.76	2.16 ± 1.19	4.52 ± 0.37
Post treatment TSH(mU/L)	3.87 ± 0.40	2.2 ± 1.2	1.35 ± 0.51	2.49 ± 0.38	1.29 ± 0.47	2.54 ± .14	2.20 ± 0.87	1.33 ± 1.12	2.93 ± 0.48
Bassline FT4 (ng/dl)	15.85 ± 1.86	16.7 ± 2.9	NR	0.35 ± 0.05	10.42 ± 1.79	12.84 ± 1.92	NR	NR	12.51 ± 2.05
Post FT4 (ng/dl)	15.70 ± 1.67	16.1 ± 3.1	NR	0.34 ± 0.05	10.99 ± 2.45	13.07 ± 2.21	NR	NR	12.25 ± 1.82
L-T4 treatment	No	NO	NR	NR	NR	NO	NO	NO	Yes
L-T4 dose	NR		NR	NR	NR		NR		89.8 ± 11.5 mg/day
Metformin dose	NR	1000 mg/day	500 mg twice daily	500 mg twice daily	2000 mg/da	NR	NR	Mean: 1747 mg/day	NR
Treatment duration (month)	6	1	6	6	6	6	12	12	6

#: NR, not reported.

*: Mean ± SD.

**Figure 3:**
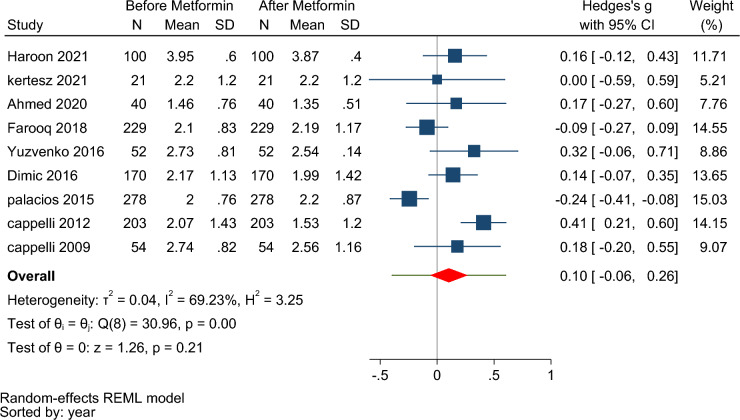
Forest plot to compare serum TSH levels before and after metformin therapy in euthyroid patients.

Pooling data under the random effect model from nine studies indicated no significant difference in TSH levels before and after taking metformin (Hedges’s g: 0.10, 95% CI  − 0.06, 0.26, *p*-value = 0.21) (Fig. 3). The subgroup analysis showed no statistically significant difference based on the duration of treatment and type of study (Fig. 1 Supplementary).

Studies were heterogeneous (I^2^ = 69.23, τ^2^ = 0.04, Q (8) = 30.96, *p* < 0.001). The funnel plot (Fig. 2 Supplementary) and the results of the begg test (z = 0.1, *p* = 0.99) indicated no publication bias.

#### Effect of metformin treatment on FT4

829 people were examined before and after intervention with metformin. The mean age of the subjects was 55.07 ± 7.73 years. The mean FT4 concentration was 11.84 ± 1.87 and 11.79 ± 1.91 ng/dl before and after the intervention, respectively.

**Figure 4:**
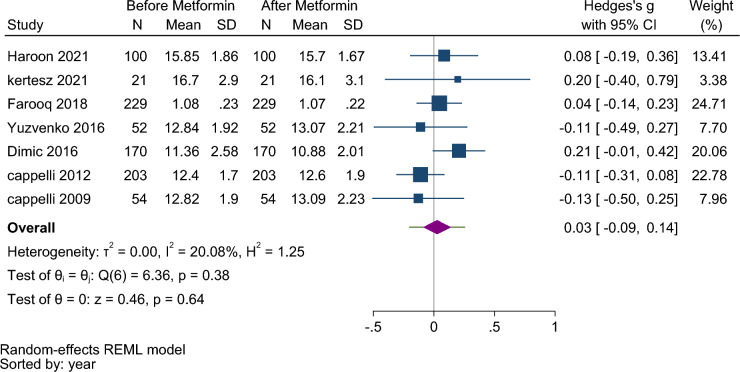
Forest plot to compare serum FT4 levels before and after metformin therapy in euthyroid patients.

Pooling data under the random effect model from 7 studies^10,11,13,15,18–20^ showed no significant difference in FT4 levels before and after taking metformin (Hedges’s g: 0.03, 95% CI  − 0.09, 0.14, *p*-value = 0.64) (Fig. 4). Therefore, the prediction interval for hedges’s was:  − 0.20, 0.25. The subgroup analysis showed no statistically significant difference based on the duration of treatment and type of study (Fig. 3 Supplementary).

The studies were homogenous (I^2^ = 20.08, τ^2^ = 0.00, Q (6) = 6.36, *p* = 0.38). The funnel plot (Fig. 4 Supplementary) and the results of the begg test (z = 0.3, *p* = 0.76) showed no publication bias.

### Hypothyroid patients

#### Effect of metformin treatment on TSH

Five studies were conducted on hypothyroid patients; four of which were clinical trial^10,12,13,20^ and one had a retrospective design^17^. Duration of metformin use was less than six months in three studies and between 6 and 24 months in two studies. Two hundred twelve people in the metformin group were evaluated. The mean age of the subjects was 54.85 ± 5.35 years. The mean TSH concentration was 2.88 ± 0.94 and 1.50 ± 0.84 mIU/L before and after the intervention, respectively (Table 2).

**Table 2:** The characteristics of hypothyroid patients under LT4 monotherapy at the baseline and follow-up.

Author, year	Haroon, 2021^10^	Theme 2021^12^	Dimick, 2016^13^	Cappelli, 2009^20^	Isidro, 2007^17^
Sample size	60	30	85	29	8
Male/female	Both	NR	NR	NR	Female
Mean age	45.2 ± 7.8		NR	NR	NR
Bassline TSH (mU/L)	3.66 ± 0.84	2.74 ± 1.13	2.17 ± 1.13	2.37 ± 1.17	3.11 ± 0.57
Post TSH (mU/L)	2.33 ± 0.70	1.27 ± 1.17	1.99 ± 1.42	1.41 ± 1.21	1.18 ± 0.36
Bassline FT4 (ng/dl)	14.96 ± 2.07	NR	11.36 ± 2.58	12.49 ± 2.09	1.23 ± 0.06
Post FT4 (ng/dl)	14.54 ± 2.06	NR	10.88 ± 2.01	12.63 ± 2.72	1.32 ± 0.04
L-T4 treatment	No	YES	NR	Yes	Yes
L-T4 dose	NR	NR	NR	89.8 ± 11.5 mg/day	1.21 ± 0.13
Metformin dose	NR	NR	2000 mg/da	NR	1700 mg daily
Treatment duration (month)	6	24	6	6	3

**Figure 5:**
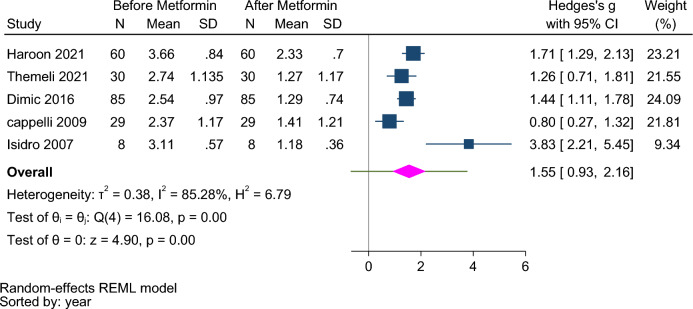
Forest plot to compare serum TSH levels before and after metformin therapy in hypothyroid patients under LT4 monotherapy.

Pooling data under the random effect model from 5 studies showed that there was a significant difference in TSH levels before and after taking metformin (Hedges’s g:1.55, 95% CI 0.93–2.16, *p*-value < 0.001) (Fig. 5). The prediction interval for hedges was:  − 0.66, 3.76 indicating a weak evidence of the effectiveness of metformin on serum TSH level in hypothyroid patients. The subgroup analysis indicated a statistically significant difference based on the duration of treatment and type of study (Fig. 5 Supplementary).

**Figure 6:**
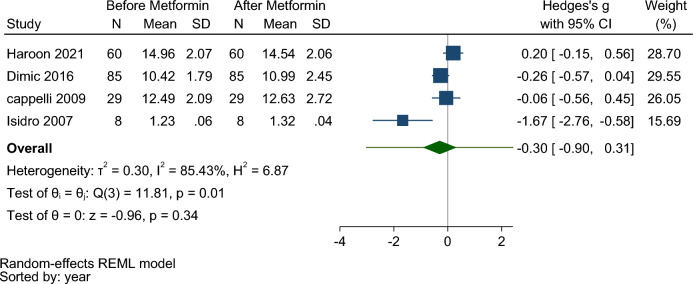
Forest plot to compare serum FT4 levels before and after metformin therapy in hypothyroid patients under LT4 monotherapy.

The studies were heterogeneous (I^2^ = 85.28, τ^2^ = 0.38, Q (4) = 16.08, *p* < 0.001). The funnel plot (Fig. 6 Supplementary) and the results of the begg test (z = 0.24, *p* = 0.80) indicated no publication bias.

#### Effect of metformin treatment on FT4

182 people were examined before and after intervention with metformin. The mean age of the subjects was 45.2 ± 7.8 years. The mean FT4 concentration was 9.77 ± 1.50 and 9.87 ± 1.81 ng/dl before and after the intervention, respectively.

Pooling data under the random effect model from 4 studies^10,13,17,20^ showed no significant difference in FT4 levels before and after taking metformin (Hedges’s g:  − 0.30, 95% CI  − 0.90, 0.31, *p*-value = 0.34) (Fig. 6). Therefore, the prediction interval for hedges was:  − 3.01, 2.42. One retrospective study showed a significant decrease in FT4 level (Hedges’s g:  − 1.67, 95% CI  − 2.76,  − 0.58, *p*-value = 0.003) (Fig. 7 Supplementary).

The studies was heterogeneous (I^2^ = 85.43, τ^2^ = 0.30, Q (3) = 11.81, *p* = 0.01). The funnel plot (Fig. 8 Supplementary) and the results of the begg test (z = 1.02, *p* = 0.73) indicated no publication bias.

## Discussion

The current meta-analysis revealed that metformin reduces serum TSH levels in patients with hypothyroidism while it has no effect on euthyroid patients' TSH levels. Moreover, metformin did not affect FT4 levels in either hypothyroid or euthyroid individuals.

Metformin's mechanism of action on the HPT axis is complex and multifactorial. Metformin may alter the affinity and/or the number of thyroid hormone receptors, increase central dopaminergic tone, or directly influence TSH regulation, augmenting the effect of thyroid hormones on the pituitary gland^21,22^. It can also increase the absorption of levothyroxine through the gastrointestinal tract, induce subtle changes in the proteins that bind to thyroid hormones, or impact thyroid hormone metabolism^17,20,21^. The central effects of metformin on TRH/TSH regulation may be mediated by the AMPK system, although its exact mechanisms are still unclear^23^. Contrary to its peripheral effects, metformin inhibits AMPK activity in the hypothalamus and increases the inhibitory effect of thyroid hormones on TSH secretion. These effects do not alter the TSH levels in the thyroid gland with normal function. Nevertheless, based on the evidence regarding TSH reduction in individuals with clinical and subclinical hypothyroidism, it is hypothesized that TSH decreases in individuals with feedback disorders in regulating the HPT axis, but not in those with a normal axis. Other studies have found no change in serum TSH levels in euthyroid people^24,25^.

Another hypothesis is that metformin stored in the body improves thyroid function in both treated and untreated hypothyroid patients.

Serum T4 levels were unaffected by metformin treatment^15,17,20,26^. Due to the lack of evidence regarding increased FT4 levels in metformin users, the hypothesis of a possible effect of metformin on increasing the gastrointestinal absorption of LT4 and resulting suppression of TSH is less relevant. The potential effects of metformin on L-T4 absorption and bioavailability do not appear to influence changes in serum TSH levels. Even though the hypothesis of the effect of metformin on the bioavailability of LT4 drug cannot be ignored, it does not appear convincing to raise this hypothesis given the evidence of the decreasing effect of metformin on TSH in other researches, even in people with subclinical hypothyroidism who do not take levothyroxine. Therefore, there is a possibility that the suppression of TSH in patients is due to a small and continuous increase in thyroid hormones that is not detectable due to an insignificant change.

There are some limitations to the current meta-analysis. First, one study was conducted only on women^17^ and there was no information on patient’s gender in three studies^12,13,20^. Second, the reviewed studies did not report serum TSH levels less than or greater than 3 mU/l, so that we could not make a comparison in these subgroups. Also, the ratio of T3 to T4 was not investigated in the studies. Finally, we could not classify and report the results in BMI subgroups due to the lack of data on BMI values.

However, future research to examine this topic needs more suitable design with an adequate sample size, and consideration of the effects of different confounding variables e.g. thyroid autoimmunity, BMI, smoking, etc.

## Conclusion

Metformin reduces serum TSH levels in hypothyroid patients. However, it has no effect on TSH levels in euthyroid individuals. In addition, metformin does not affect serum FT4 levels in euthyroid or hypothyroid individuals. TSH reducing effect of metformin necessitate clinicians to be more cautious when adjusting LT4 dosage in diabetic patients to avoid hyperthyroidism due to overtreatment especially in the elderly.

### Supplementary Information

Supplementary Information.

## Data Availability

The datasets used and/or analysed during the current study available from the corresponding author on reasonable request.
