# Supplementary material for: The effect of metformin therapy on serum thyrotropin and free thyroxine concentrations in patients with type 2 diabetes: a meta-analysis
Source: Sci Rep. 2023 Oct 31;13:18757. doi: 10.1038/s41598-023-43266-9 (PMC10618453; doi:10.1038/s41598-023-43266-9)
Supplement: Supplementary file 1 — Supplementary Information. [file 41598_2023_43266_MOESM1_ESM.docx]

Fig 1 supplementary: Subgroup analysis for comparing serum TSH level before and after metformin therapy euthyroid patient-based LT4, type study and duration treatment

Fig 2 supplementary: Funnel plots of Hedges’s’g effect sizes for TSH lead level at the euthyroid patient

Fig 3 supplementary: subgroup analysis for comparing serum FT4 level before and after metformin therapy in euthyroid patient-based LT4, type study and duration treatment

Fig 4 supplementary: Funnel plots of Hedges’s’g effect sizes for FT4 lead level at the euthyroid patient

Fig5 supplementary: subgroup analysis for comparing TSH level in before and after metformin group at hypothyroid patient-based LT4, type study and duration treatment

Fig 6 supplementary: Funnel plots of Hedges’s’g effect sizes for TSH lead level at hypothyroid patients

Fig 7 supplementary: subgroup analysis for comparing FT4 level in before and after metformin group at hypothyroid patient-based LT4, type study and duration treatment

Fig 8 supplementary: Funnel plots for risk of bias of Hedges’s’g effect sizes for FT4 lead level at the hypothyroid patient

supplementary appendix

**Scopus**

( TITLE-ABS-KEY ( hypothyroidism ) OR TITLE-ABS-KEY ( hyperthyroidism ) OR TITLE-ABS-KEY ( "subclinical hypothyroidism" ) OR TITLE-ABS-KEY ( "subclinical hyperthyroidism" ) OR TITLE-ABS-KEY ( "thyroid hormons" ) OR TITLE-ABS-KEY ( "thyroid dysfunction" ) OR TITLE-ABS-KEY ( "thyroid simulating" ) OR TITLE-ABS-KEY ( thyroxine ) OR TITLE-ABS-KEY ( "free thyroxin*" ) OR TITLE-ABS-KEY ( tsh ) AND TITLE-ABS-KEY ( metformin ) OR TITLE-ABS-KEY ( biguanide ) AND TITLE-ABS-KEY ( "diabetes mellitus" ) OR TITLE-ABS-KEY ( "type 2 diabetes" ) OR TITLE-ABS-KEY ( "type2 diabetes" ) OR TITLE-ABS-KEY ( niddm ) )

**PubMed**

**(((((((((((hypothyroidism[Title/Abstract]) OR (hyperthyroidism[Title/Abstract])) OR (thyroxin[Title/Abstract])) OR ("thyroid dysfunction"[Title/Abstract])) OR ("thyroid simulating"[Title/Abstract])) OR ("thyroid hormon*"[Title/Abstract])) OR ("free thyroxin*"[Title/Abstract])) OR ("subclinical hypothyroidism"[Title/Abstract])) OR ("subclinical hyperthyroidism"[Title/Abstract])) OR (TSH[Title/Abstract])) AND (((("diabetes mellitus"[Title/Abstract]) OR ("type 2 diabetes"[Title/Abstract])) OR ("type2 diabetes"[Title/Abstract])) OR (NIDDM[Title/Abstract]))) AND (((metformin[Title/Abstract]) OR (biguanide[Title/Abstract])))**

**ISI**

**TI= (hypothyroidism OR hyperthyroidism OR thyroxin OR "thyroid dysfunction" OR "thyroid simulating" OR "thyroid hormone*” OR "free thyroxin*" OR "subclinical hypothyroidism" OR "subclinical hyperthyroidism" OR TSH) AND ("diabetes mellitus" OR "type 2 diabetes” OR "type2 diabetes" OR NIDDM) AND (Metformin OR biguanide)**
